# Detecting Features of Interpersonal Difficulties in First-Person Accounts of Schizophrenia; Automated Linguistic and Network Analyses

**DOI:** 10.1093/schizbullopen/sgag017

**Published:** 2026-04-30

**Authors:** Hyeon-Seung Lee, Katrina S Rbeiz, Yunlai Gui, Kathryn Babbitt, Tatiana Baxter, Michael Sangimino, Pietra Bruni, Anne Felsenheimer, Olivia Jelsma, Mingyuan Li, Brian Kim, Adi Peres, James Yang, Sohee Park

**Affiliations:** Department of Psychology, Vanderbilt University, Nashville, TN 37240, United States; Department of Psychology, Vanderbilt University, Nashville, TN 37240, United States; Department of Psychology, Vanderbilt University, Nashville, TN 37240, United States; Department of Psychology, Vanderbilt University, Nashville, TN 37240, United States; Department of Psychology, Vanderbilt University, Nashville, TN 37240, United States; Department of Psychology, Vanderbilt University, Nashville, TN 37240, United States; Department of Psychology, Vanderbilt University, Nashville, TN 37240, United States; Department of Psychology, Vanderbilt University, Nashville, TN 37240, United States; Department of Psychology, Vanderbilt University, Nashville, TN 37240, United States; Department of Psychology, Vanderbilt University, Nashville, TN 37240, United States; Department of Psychology, Vanderbilt University, Nashville, TN 37240, United States; Department of Psychology, Vanderbilt University, Nashville, TN 37240, United States; Department of Psychology, Vanderbilt University, Nashville, TN 37240, United States; Department of Psychology, Vanderbilt University, Nashville, TN 37240, United States

**Keywords:** schizophrenia, schizotypy, interpersonal difficulties, phenomenology, narratives

## Abstract

**Background:**

First-person accounts (FPAs) provide multi-faceted insights into the lived social experiences of schizophrenia. We examined FPAs and third-person accounts (TPAs) using automated linguistic and network analyses to extract narrative patterns in relation to interpersonal difficulties.

**Study Design:**

Narratives from 279 articles published in *Schizophrenia Bulletin* (1979-2025) were screened to identify 133 FPAs and 44 TPAs. Schizotypal Personality Questionnaire was used to assess syndromes of schizotypy expressed in the narratives. At least one cognitive-perceptual syndrome (ideas of reference, odd beliefs, magical thinking) was detected in every FPA (*n* = 133). Interpersonal difficulties (social anxiety, no close friends, constricted affect) were detected in 56 FPAs and designated as the “interpersonal difficulties” group. We used the Linguistic Inquiry and Word Count software to extract interpersonally relevant features: authenticity, emotional tone, pronoun use, social behavior, and confidence. Linguistic features of narratives in FPAs with and without interpersonal difficulties were compared using network analysis.

**Study Results:**

Greater authenticity and lower confidence were detected in FPAs compared to TPAs. FPAs contained fewer social words and more “I” words than TPAs. FPAs with interpersonal difficulties were longer, more negative, and less linguistically formal than FPAs without interpersonal difficulties. Network analysis indicated a link between interpersonal difficulties and “voices.”

**Conclusions:**

Narratives categorized by interpersonal difficulties exhibit distinct linguistic signatures revealed through automated linguistic tools and network analyses. Despite the vast range of topics in FPAs, these computational methods provide a robust quantitative framework for validating the lived experience of negative syndrome in schizotypy.

## Introduction

Schizophrenia (SZ) is a severe and debilitating condition, characterized by psychotic symptoms along with social impairment and withdrawal.^1^ Deterioration of social functioning, present from the premorbid stage and throughout the course of the illness, predicts poor outcome in SZ.^2^^,^^3^ Loss of social opportunities due to symptoms can lead to chronic social isolation, which further erodes interpersonal relationships, forming a vicious cycle of disconnection, defeat, and disability in this population.^4–6^ This persistence of social impairments creates a major barrier to recovery with a heavy personal and societal toll. Thus, understanding the nature of these social impairments to develop effective treatments is an important goal for clinical science and society.

Past research indicates that individuals with SZ-spectrum conditions are 6 times more likely to experience interpersonal difficulties compared to healthy individuals.^7–9^ Furthermore, nearly half of individuals with SZ express a need for more friends, highlighting the acute sense of social isolation they endure.^9^ Thus, loneliness is a significant aspect of their experience, identified as the second greatest challenge after financial concerns.^10^ Despite the importance of the impact of interpersonal difficulties on individuals with SZ, the subjective experience of disconnection has been relatively neglected in this population,^11^ and personal accounts by individuals with SZ tend to be minimized during standardized interviews.^12–14^ Given the uniqueness and richness of personal experiences and their implications for treatment outcomes, the viewpoints of each individual should be considered with care and attention.^12^^,^^15–17^

Written first-person narratives provide an excellent opportunity to gain insight into the lived experience of psychosis that might not be disclosed in clinical interviews or self-report questionnaires, freed from the structural confines of hospital or research settings. From first-person accounts (FPAs), it is possible to extract the personal and existential dimensions of living with SZ, and several previous studies have investigated whether a particular pattern of language use might be associated with specific symptoms and experiences. For example, linguistic analysis has revealed differences in word category use by different diagnostic groups.^17^ It has also been possible to identify meaningful constructs that predict recovery from context analysis and manual thematic coding of narratives.^12^

Previous studies of narratives have mostly used qualitative methods.^18–20^ While qualitative analysis reveals key aspects of salient subjective experiences, it is also susceptible to raters’ biases and can be burdensome.^12^^,^^17^ Alternatively, computational language analysis tools have been used with some success in diagnostic classification^21–23^ and symptom identification beyond clinical ratings.^24^^,^^25^ Therefore, in the current study, we applied automated linguistic tools to extract clinically relevant information about interpersonal difficulties from a corpus of written narratives published in *Schizophrenia Bulletin*.

Taken together, the current study aimed to examine interpersonal difficulties in individuals with SZ using a quantitative analysis of FPAs. FPAs of SZ spectrum conditions published in the journal, *Schizophrenia Bulletin* since 1979, provide a rich and accessible collection of illness narratives of people with lived experiences as well as third-person accounts (TPAs) by family members or mental health professionals. We compared the FPAs and TPAs of SZ published in *Schizophrenia Bulletin* using an automated linguistic analysis tool, Linguistic Inquiry and Word Count (LIWC) software^26^^,^^27^ expanding on a previous study.^12^ We hypothesized that FPAs would be linguistically distinct from TPAs, such that FPAs would report lower social confidence and less frequent use of social words or pronouns. Furthermore, we hypothesized that within the FPAs, narratives reporting interpersonal difficulties would show a unique linguistic profile compared to those without, such as a higher negative tone. Lastly, we hypothesized that our exploratory word network analysis would reveal central themes specific to the experience of interpersonal difficulties in FPAs.

## Methods

### Narrative Search, Screening, and Review

We identified 279 narratives published in *Schizophrenia Bulletin* between 1979 and April of 2025. Two groups of independent raters conducted a screening of these 279 narratives to obtain accounts of people with lived experiences of psychosis. Narratives consisting of poems, artistic expression (*n* = 6), and those written by people with other psychopathology (bipolar, autism spectrum, obsessive compulsive, and major depressive disorders, *n* = 7) were excluded because they were beyond the scope of this study. Among the 279 narratives, 112 narratives were written by 23 authors. For instance, 30 narratives were written by one author (Jason A. Jepson). We combined articles written by the same author, resulting in 177 narratives. The topics of the narratives included onset of illness, mental illness struggles, stigma, treatment, hospitalization, and the ways in which psychosis affected the authors’ family members, social relationships, careers, and daily lives.

**Figure 1:**
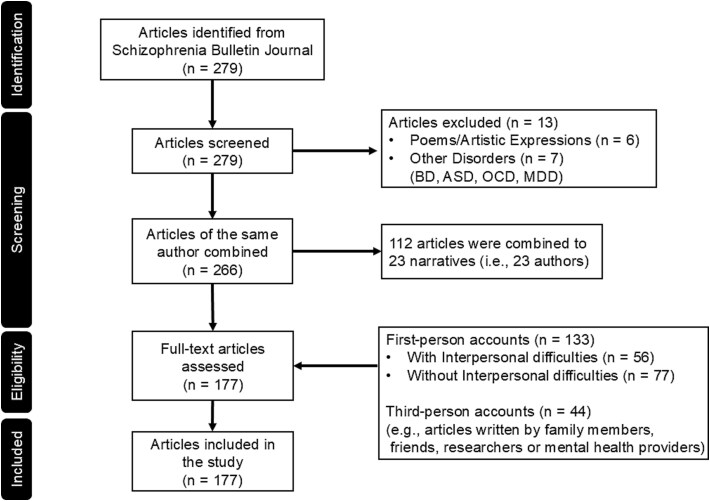
Selection Criteria and Process for the First-Person and Third-Person Accounts.

Qualitative reviews were conducted by the authors to identify TPAs written by family members, friends, and clinicians (eg, psychologists, medical doctors, nurses, etc.). There were 133 FPAs written by individuals with SZ and 44 TPAs (see Figure 1). After an initial review, 3 raters conducted a cross-check to reach consensus. See Table S1 for the review results across each narrative.

Oxford Academic, the publishers of the *Schizophrenia Bulletin*, allows text and data mining of their publications, without the need for formal permission, if the data are used for “non-commercial purposes.”^12^

### Categorizing the FPAs

We aimed to identify evidence of clinical syndromes as expressed in the narratives. Given that FPAs do not always provide the specific diagnoses, we used the Schizotypal Personality Questionnaire (SPQ^28^) to train the raters to guide the detection and identification of schizotypal syndromes (eg, cognitive-perceptual and interpersonal symptoms) in the narratives: ideas of reference, social anxiety, magical thinking, unusual perceptual experiences, eccentric/odd behavior, no close friends, odd speech, constricted affect, and suspiciousness. This strategy allowed us to include less severe symptoms such as magical thinking, unusual perceptual experiences, and interpersonal difficulties reported by people with SZ spectrum conditions. All raters entered their results to a common spreadsheet, and the 3 lead authors (H.-S.L., K.S.R., Y.G.) met as a group to reach consensus per narrative. All the FPAs reviewed mentioned at least one cognitive-perceptual symptom (ie, ideas of reference, magical thinking, unusual perceptual experiences, suspiciousness) (*n* = 133). Reviewers also extracted sentences mentioning interpersonal difficulties using the following SPQ subscales: no close friends, excessive social anxiety, constricted affect (see Table S3). The interrater reliability using Krippendorff’s α = .82, 95% CI, 0.74-0.88 indicated good agreement. The 3 lead authors coded each narrative, and discrepancies were resolved by consensus. Among 133 FPAs, 56 narratives contained language consistent with the SPQ interpersonal-symptom domains and were designated as the “interpersonal difficulties” group. For instance, Anonymous (1994)^29^ reported, “I want friends, but I don’t know how to make them.” Gardiner (2021)^30^ reported, “I am locked in my little room alone for 22 hours a day.” DeMann (1994)^31^ reported, “I started to withdraw socially.” Such descriptions of interpersonal difficulties were identified and counted per each FPA. Notably, the prolific authors, who each wrote multiple narratives, repeatedly mentioned interpersonal difficulties throughout their works, rather than limiting it to a single narrative (see Tables S1 and S2).

### The LIWC Program and Network Analysis

The LIWC was used to analyze the narratives.^26^^,^^27^ LIWC is a text analysis tool that quantifies written texts based on calculations of the proportion of words falling into specific categories relative to the total word count. LIWC outputs a set of “basic” and “summary” variables that provide insights into the content and structure of the analyzed text.^26^^,^^27^ The “summary” variables offer a condensed overview of the text’s characteristics including Authenticity (perceived honesty or genuineness of the narrative), Clout (narrator’s perceived social status, confidence, or leadership), and Analytic (formal, logical, or hierarchical thinking patterns). Given that these variables provide high-level linguistic information about the narrator’s psychological and cognitive style, we included all 3 in our analyses. In addition, we sought to investigate more specific linguistic features. Previous studies indicate that specific pronoun use (eg, “I” or “we”) is associated with interpersonal difficulties.^25^^,^^32^^,^^33^ Furthermore, examining the emotional valence in accounts can provide further insight into the authors’ appraisal of personal experience. Therefore, we included 4 “basic” variables: Total Pronouns (both impersonal and personal pronouns usage), Positive Tone (positive sentiment/affect), Negative Tone (negative sentiment/affect), and Social (a broad set of social behaviors and interpersonal communication). Then, specific pronoun use such as “I,” “we,” “you,” “she/he,” and “they” was also compared between groups.

We also conduct word-level network analysis for all the narratives to investigate contextual co-occurrence of words and to explore word-level connections. The nodes of words connected to one another are visualized as a result of network analyses, as done in previous studies utilizing speech graph analysis.^21–23^^,^^34^

### Data Analysis

Independent *t*-tests were conducted between FPAs and TPAs on 3 summary (Authentic, Clout, and Analytic) and 4 basic (Positive tone, Negative tone, Social, and Total pronouns) LIWC variables. Since LIWC emotional tone scores are determined by dictionary-matched word ratio, the presence of negated emotions (eg, not good) can result in inverted or inaccurate valence scoring. To ensure the purity of the emotional tone metrics, we identified instances of negation across groups (9 cases in TPAs, and 13 and 15 cases in individuals with SZ with and without interpersonal difficulties, respectively). These negated emotional expressions were excluded from the final tone analysis to verify that the results reflected the true emotional valence of the narratives, independent of linguistic artifacts. In addition to LIWC variables, specific pronoun use, such as “I,” “we,” “you,” “she/he,” and “they,” was also compared between groups. When Levene’s test indicated a violation of the assumption of homogeneity of variances, Welch’s *t-*test was used. To reduce type I errors from multiple comparisons with maintaining enough power, the Benjamini-Hochberg false discovery rate method was applied to *P*-values.^35^

Word count was significantly larger in the FPAs with interpersonal difficulties (Mean = 4064.05, SD = 4580.68) than those without interpersonal difficulties (Mean = 2021.91, SD = 1170.98) (*t*_60.25_ = 3.26, *P* < .001^**^, *d* = 0.61). We conducted analyses of covariance with word count as covariate. Word count was not a significant covariate for all LIWC and pronoun variables (all *P* > .11), and the findings did not show inconsistency. Thus, we utilized an independent *t*-test to compare between FPAs.

Also, correlational analyses were conducted between the frequency of reporting interpersonal difficulties, LIWC variables, and specific pronouns. Partial correlation controlling word counts was conducted.

Moreover, a word network analysis was conducted to map word frequencies and relationships within the narratives, utilizing the igraph package in R (version 4.2.3).^36^ For each group (eg, all FPAs, TPAs), texts were aggregated and preprocessed, excluding stop words and non-meaningful words (eg, “things,” “one,” “will,” and “much”). The nodes in each network represent the top 20 most frequent words for that group. Connections (edges) were established based on word co-occurrences within the narratives, with weights determined by the frequency of these co-occurrences. Crucially, the size of each node and its corresponding label were scaled by Betweenness Centrality, a metric that captures the extent to which a node serves as a “bridge” between separate semantic clusters that lack a direct connection. These nodes represent the key connectors that fill the gaps between different semantic domains.

We retained the full network structure for centrality calculations but applied a grayscale transparency (alpha gradation) to the edges such that the top 40% are highlighted with weaker links rendered transparently. This ensures that the visual prominence of nodes accurately reflects their structural importance across the entire network, including the numerous lower-weight connections that facilitate their central bridging roles. The word network was analyzed for FPAs, TPAs, and FPAs with and without interpersonal difficulties, respectively.

## Results

### Comparison Between FPAs and TPAs Using LIWC Variables

Word count was not significantly different between FPAs (Mean = 2881.76, SD = 3249.08) and TPAs (Mean = 2669.73, SD = 1629.20) (*t* = 0.42, *P_adj_* = .69, Cohen’s *d* = −0.08).

First, FPAs (*n* = 133) were evaluated as more Authentic (*t*_60.48_ = 10.61, *P_adj_* < .001^**^, *d* = 1.96) and having lower Clout (*t*_59.10_ = −11.52, *P_adj_* < .001, *d* = −2.15) in comparison to TPAs (*n* = 44) (see Table 1). The findings suggest that FPA of SZ individuals exhibit greater self-disclosure and lower confidence compared with TPAs (written by a family member, a friend, or a mental health professional). Furthermore, FPAs contained fewer social words (ie, words indicating social behaviors and interpersonal communication) compared to TPA (*t*_175_ = −12.30, *P_adj_* < .001^**^, *d* = −2.03). There was no significant difference in total pronoun use between FPA and TPA (*t*_59.61_ = 1.04, *P_adj_* = .42, *d* = 0.19); however, “I” word use was greater in FPA (*t*_175_ = 8.60, *P_adj_* < .001^***^, *d* = 1.52), while “We” (*t*_53.90_ = −5.26, *P_adj_* < .001^***^, *d* = −1.02) and “She/He” (*t*_47.10_ = −9.01, *P_adj_* < .001^***^, *d* = −1.84) pronoun use was greater in TPA (Table 2). “You” (*t*_175_ = 0.92, *P_adj_* = .45, *d* = 0.19) and “They” words (*t*_175_ = 0.45, *P_adj_* = .66, *d* = 0.08) use was comparable between FPA and TPA.

**Table 1:** Linguistic inquiry and word count program (LIWC) summary variables.

**LIWCS variables**	**First-person account (FPA) of SZ (*n* = 133)** M (SD*)*	Third-person account (TPA) (*n* = 44) M (SD)	Test statistics (*t, P_adj_, Cohen’s d*)
Authentic	85.34 (18.36)	43.37 (24.02)	***t* = 10.61, *P***_***adj***_ **<.001, *d* = 1.96**
Clout	11.30 (16.83)	54.49 (22.92)	***t* = −11.52, *P***_***adj***_ **<.001, *d* = −2.15**
Analytic	50.07 (17.65)	53.66 (20.09)	*t* = −1.13, *P_adj_* = .42, *d* = −0.19
Positive tone	2.65 (0.97)	2.59 (0.76)	*t* = 0.36, *P_adj_* = .78, *d* = 0.07
Negative tone	2.32 (0.85)	2.36 (0.81)	*t* = −0.28, *P_adj_* = .78, *d* = −0.05
Social	8.38 (2.76)	14.63 (3.36)	***t* =** −**12.30, *P***_***adj***_ **<.001, *d* =** −**2.03**
Total pronouns	15.71 (2.93)	15.04 (3.94)	*t* = 1.04, *P_adj_* = 0.42, *d* = 0.19
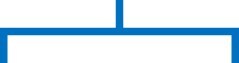			
**LIWCS variables**	**SZ with interpersonal difficulties (*n* = 56) M (SD)**	**SZ w/o interpersonal difficulties (*n* = 77) M (SD)**	**Test statistics (*t, P_adj_, Cohen’s d*)**
Authentic	87.94 (15.23)	83.45 (20.22)	*t* = 1.46, *P_adj_* = .39, *d* = 0.25
Clout	9.65 (15.05)	12.50 (18.02)	*t* = −0.96, *P_adj_* = .20, *d* = −0.17
Analytic	45.56 (14.96)	53.36 (18.79)	***t* =** −**2.57, *P***_***adj***_ **= .028, *d* = 0.46**
Positive tone	2.42 (0.77)	2.82 (1.06)	***t* =** −**2.55, *P***_***adj***_ **= .028, *d* =** −**0.42**
Negative tone	2.53 (0.80)	2.17 (0.86)	***t* = 2.41, *P***_***adj***_ **= .030, *d* = 0.43**
Social	8.36 (2.72)	8.40 (2.81)	*t* = −0.09, *P_adj_* = .93, *d* = 0.02
Total pronouns	16.53 (2.63)	15.11 (3.01)	***t* = 2.83, *P***_***adj***_ **= .028, *d* = 0.50**

Authentic, clout, Analytic summary variables are algorithms derived from accumulated empirical data (see https://www.liwc.app/). The numbers are standardized scores that have been converted to percentiles.

“Authentic” captures the degree of spontaneity, genuineness, or reduced filtering/monitoring in the writing.

“Clout” captures relative social status, confidence, or leadership that the person displays in the writing.

“Analytic” captures formal, logical, and hierarchical thinking patterns.

“Positive” and “negative tones” are computed as the percentage of valence-matched words relative to the total word count.

“Social” variable is computed as the percentage of social process words (words referencing social relationships and behaviors) relative to the total word count.

“Total Pronoun” is the combined percentage of all pronouns.

*P_adj_*: False discovery rate corrected *P*-value by Benjamini-Hochberg method.

*t*: the value of the t-statistic from the group comparison test

*d*: Cohen’s D effect size

Abbreviation: SZ, schizophrenia.

**Table 2:** Pronoun use in the FPAs of SZ with or without interpersonal difficulties and in the TPAs.

**Pronouns**	**First-person account (FPA) of SZ (*n* = 133)** M (SD)	Third-person account (TPA) (*n* = 44) M (SD)	Test statistics (*t, P_adj_, Cohen’s d*)
I	8.53 (2.86)	4.30 (2.72)	***t* = 8.60, *P***_***adj***_ **<.001, *d* = 1.52**
We	0.36 (0.56)	1.14 (0.93)	***t* =** −**5.26, *P***_***adj***_ **<.001, *d* =** −**1.02**
You	0.39 (0.94)	0.26 (0.35)	*t* = 0.92, *P_adj_* = .45, *d* = 0.19
She/He	0.73 (1.01)	4.45 (2.67)	***t* =** −**9.01, *P***_***adj***_ **<.001, *d* =** −**1.84**
They	0.75 (0.52)	0.72 (0.41)	*t* = 0.45, *P_adj_* = .66, *d* = 0.08
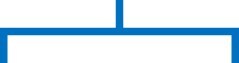			
**Pronouns**	**SZ with interpersonal difficulties (*n* = 56) M (SD)**	**SZ w/o interpersonal difficulties (*n* = 77) M (SD)**	**Test statistics (*t, P_adj_, Cohen’s d*)**
I	9.22 (2.59)	8.02 (2.96)	*t* = 2.43, *P_adj_* = .08, *d* = 0.43
We	0.37 (0.59)	0.35 (0.55)	*t* = 0.15, *P_adj_* = 1.00, *d* = 0.03
You	0.32 (0.43)	0.44 (1.18)	*t* = −0.77, *P_adj_* = .74, *d* = −0.14
She/He	0.84 (1.12)	0.65 (0.92)	*t* = 1.07, *P_adj_* = .72, *d* = 0.19
They	0.74 (0.39)	0.76 (0.60)	*t* = −0.21, *P_adj_* = 1.00, *d* = −0.04

Pronoun use scores were computed as the percentages of the total word count.

*P_adj_*: False discovery rate corrected *P*-value by Benjamini-Hochberg method.

*t*: the value of the t-statistic from the group comparison test

*d*: Cohen’s D effect size

Abbreviation: SZ, schizophrenia.

### Comparisons Between FPAs With or Without Interpersonal Difficulties

We examined the role of interpersonal difficulties in LIWC variables. As already reported above in the “Data Analysis” section, the absolute word count was twice as large in FPAs with interpersonal difficulties than in FPAs without interpersonal difficulties (*t*_60.25_ = 3.26, *P_adj_* < .001^**^, *d* = 0.61) and therefore word count was covaried out for all the analyses reported below.

First-person accounts reporting interpersonal difficulties (*n* = 56) showed lower Analytic scores than FPAs without interpersonal difficulties (*n* = 77) (*t*_131_ = −2.57, *P_adj_* = .28^*^, *d* = 0.46). These results suggest that interpersonal difficulties are associated with less formal and less logical language use. FPAs reporting interpersonal difficulties had significantly less Positive Tone (*t*_130.85_ = −2.55, *P_adj_* = .28^*^, *d* = −0.42) and higher Negative Tone (*t*_131_ = 2.41, *P_adj_* = .30^*^, *d* = 0.43) than those without interpersonal difficulties. Also, SZ reporting interpersonal difficulties used pronouns more often (*t*_131_ = 2.83, *P_adj_* = .28^*^, *d* = 0.50) than SZ without interpersonal difficulties. Interestingly, there was no significant difference between the 2 groups in social word use.

### Correlation Analyses Between Interpersonal Difficulties and LIWC Variables

Partial correlations controlling word counts were conducted between the frequency of reporting interpersonal difficulties and LIWC variables. There were no significant correlations between LIWC variables and specific pronouns (all *P_adj_* > .66).

### Word Network Analysis Across FPAs and TPAs

**Figure 2:**
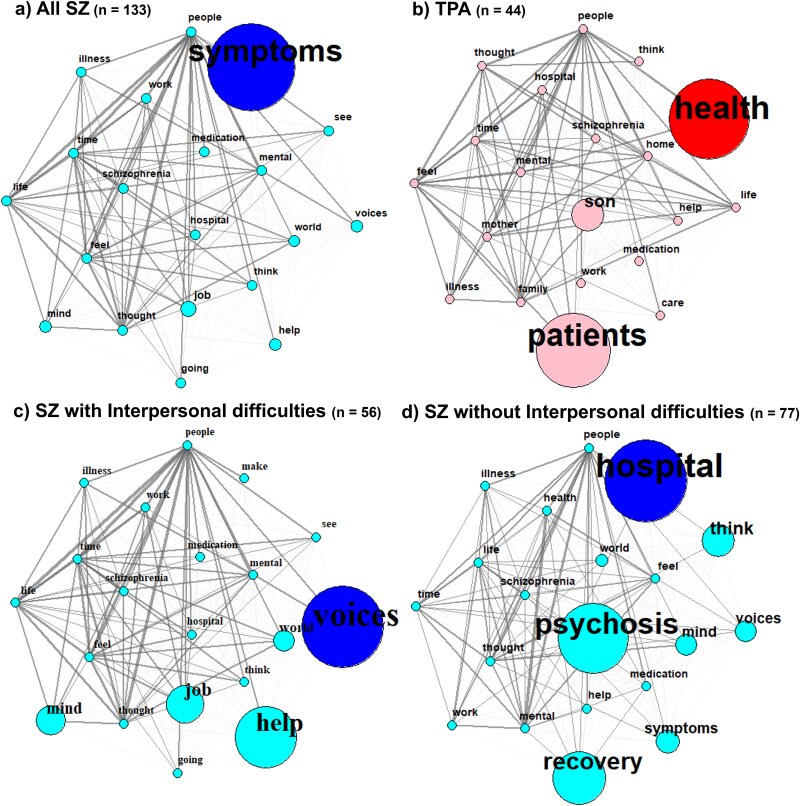
Network Analyses of First-Person and Third-Person Narratives.

Across all FPAs, the word network exhibited the word “symptoms” as the most central node (ie, having the highest betweenness centrality) (see Figure 2). The word “symptoms” was connected to the “people” node, which was connected to nodes such as “help,” “medication,” “hospital,” and “life.” These represent the ways in which psychosis symptoms affect the authors involved, including impacting their social relationship and their daily lives. It is notable that assistance and treatment seeking (including medication) were central themes of the FPAs overall.

For TPAs, the words “health” and “patients” were observed as the most central nodes in the network analysis. The word “health” was connected to the “mental” and “people” nodes. The word “patient” was connected to the nodes, “help” and “hospital,” which are closely connected to the node “schizophrenia.” These links between nodes suggest that the diagnosis and health status were central themes of the TPAs overall.

### Word Network Analysis in FPAs With or Without Interpersonal Difficulties

The words “hospital,” “psychosis,” and “recovery” were the most central nodes among the FPAs without interpersonal difficulties. On the other hand, the FPAs reporting interpersonal difficulties revealed “voices” as the most central node. This may indicate that SZ without interpersonal difficulties report psychotic symptoms and their recovery process in general, while those with interpersonal difficulties report psychotic symptoms in the form of auditory hallucinations, which may be a key “bridge” theme closely related to their experience of interpersonal difficulties. This finding is interesting in the context of the social deafferentation hypothesis and how it is represented empirically.^37–39^ According to Hoffman’s hypothesis, social isolation and disconnection can lead to hallucinations after a period of deprivation, which may lead to neural reorganization (ie, hyperactivity of the social brain network) that corresponds to hallucinated experience of others.^37^ Although the FPAs in this study does not directly address this issue, it is nevertheless noteworthy that socially disconnected individuals with SZ tended to hear “voices.”

## Discussion

This study examined lived experience of interpersonal difficulties in individuals affected by SZ-spectrum conditions by conducting an automated linguistic analysis of their written narratives. These accounts were published in the journal, *Schizophrenia Bulletin*, and they were not solicited for a specific theme.

To further analyze the association between schizotypal syndromes and language use, we categorized narratives by using the SPQ to guide the identification of syndrome.^28^ All the FPAs (*n* = 133) reported at least one positive (ie, cognitive-perceptual) syndrome. Fifty-six FPAs reported negative (ie, social withdrawal/interpersonal) syndromes. Individuals with SZ and their associates (eg, family members, friends, mental health workers) used a similar number of words in their accounts. Compared with TPAs, FPAs were associated with greater self-disclosure, lower confidence, and fewer social words. Within SZ, those with interpersonal difficulties exhibited reduced positive tone, greater negative tone, less formal, and logical language use than those without interpersonal difficulties, even after co-varying for the total word count. The total pronoun use did not differ between the FPAs and TPAs, but “I” word use was increased in FPAs. Within SZ, those reporting interpersonal difficulties used more pronouns than those without interpersonal difficulties. Lastly, word network analyses suggested that voice-hearing (ie, auditory hallucinations) was increased in SZ with interpersonal difficulties than SZ without interpersonal difficulties.

Automated language analyses showed that FPAs were evaluated as more authentic, but with less clout and contained fewer social process words than TPAs. Given that FPAs primarily centered on self-disclosed life experiences shaped by symptoms and treatments, these findings may reflect their perceived social defeat and withdrawal during the course of illness. Previous research suggested that perceived social defeat represents an individual’s negative perception or appraisal of their standing in the world.^40–42^ While FPAs were often written by individuals with chronic SZ, many of whom were in remission and had experienced at least partial treatment success, reduced self-confidence and perceived social defeat remain a significant barrier.

The first-person singular pronoun (*I*) was used more often in FPAs, while first-person plural (*we*) and third-person pronouns (*he/she/they*) were used more often in the TPAs. We acknowledge that this distribution primarily reflects the structural nature of the narratives; FPAs are inherently autobiographical, while TPAs, authored by family and professionals, naturally focus on another person. However, the observed preponderance of singular “I” over plural “we” in FPAs warrants cautious consideration in light of existing literature. Previous studies have indicated that individuals with SZ often identify themselves as a single character in their stories rather than as part of a larger collective or community.^25,32^ Furthermore, increased use of the first-person singular pronouns has been reported in the life narratives^43^ and emotion narratives^44^ of individuals with SZ relative to controls. Future research employing healthy first-person narrative controls may be necessary to further disentangle these effects.

Notably, the potential link between the increased use of first-person pronouns and interpersonal difficulties could further extend to difficulties in social functioning in SZ. For example, Buck and Penn found that elevated first-person singular pronoun use was correlated with external attribution style and poor theory of mind, both of which might be precursors for social dysfunction.^44^ Therefore, increased first-person pronoun use and its associations with interpersonal difficulties and social functioning in SZ need to be further studied in the future.

In addition to previous research that only compared narratives by SZ with controls, we further used interpersonal difficulties as a trans-diagnostic symptom and compared narratives within SZ, following the criteria of the SPQ. Dimensionally, the frequency of mentioning interpersonal difficulties after controlling for word count was not associated with the LIWC variables. However, categorically, lower Analytic scores in SZ reporting interpersonal difficulties indicate less formal and logical use of language than SZ without interpersonal difficulties. According to the LIWC manual, Analytic score is associated with executive functioning, cognitive control, structured thinking, all of which are aspects of working memory capacity that are primarily associated with prefrontal cortex activity.^27^ Therefore, lower Analytic score in SZ with interpersonal difficulties might indicate a lower level of working memory capacity. Indeed, previous studies consistently indicated the close link between severe negative symptoms (eg, avolition, anhedonia, social withdrawal) and greater working memory impairments,^45–47^ which have a critical impact on managing daily tasks and overall functioning.^48^ Though replication is required, the current finding may suggest that linguistic markers from individuals reporting interpersonal difficulties could potentially serve as an indicator of impaired cognitive functioning and its associations to specific symptoms in SZ.^49,50^

Word network analyses revealed that in narratives from FPAs reporting interpersonal difficulties, the word “voices” emerged as the most central theme (highest betweenness centrality), acting as a key structural bridge for their narrative. Interestingly, those FPAs reported that hearing voices happened especially when they are alone.^51,52^ For example, Beyer reported “When I listen to music or zoom or talk to my boyfriend, I am usually free from voices. But when I am alone in my room, I suffer, especially when there is or was some trouble. When I am all alone in my room all the time, I hear voices.”^51^ The voices often came from people the authors already knew (eg, friends,^53^ mother^54^) or God,^55^ and contained either a positive^56,57^ or negative tone.^53,57^ These align with the social deafferentation hypothesis,^37^ which posits that a loss of social connections can lead to a compensatory over-activation of the social brain network, which can generate social hallucinations and delusions (eg, hearing voices, sensed presence experience).

There are caveats. First, FPAs from *Schizophrenia Bulletin* may not be broadly representative. These authors are self-selected individuals, often in remission, with access to treatment and the motivation to submit an essay. Furthermore, the retrospective nature of these published essays inherently shapes the narratives. Secondly, the narratives were likely edited prior to publication. For example, authors often acknowledge pre-submission assistance from family, friends, or mentors, introducing potential biases.^17^ Consequently, these findings must be interpreted with caution, as their generalizability, both to the broader, heterogeneous SZ-spectrum population and to other forms of spontaneous writing, is not fully established. Thirdly, the “interpersonal difficulties” category has qualitative origins and therefore contains subjectivity, both in the inclusion/exclusion criteria set by the lead authors and in the construct’s definition itself. All subsequent analyses using LIWC and network comparisons are therefore contingent on these qualitative coding decisions. To minimize such variance, we trained coders, double-coded, reached consensus, and established good interrater reliability. Future studies need to mitigate these risks.

Previous research examining FPAs from the *Schizophrenia Bulletin* already provides interesting findings. Several studies have analyzed language use patterns in FPAs to better understand cognitive disturbances and recovery (eg, Green and García-Mieres, 2022).^12^ Our current network analysis findings also imply narrative themes and their relationships drawn from personal reflections on past symptoms and lived experiences. Furthermore, research on personal blogs argues that such narratives offer significant freedom for personal expression, providing insight into the author’s core characteristics.^58^ Though not identical to blogs, published FPAs share this quality, as individuals with SZ freely share and reflect on their personal experiences. We therefore believe these accounts offer a diverse sampling of narratives. Lastly, none of these narratives were solicited to examine interpersonal difficulties. The fact that we detected signs of isolation in free narratives, rather than through explicit questioning, strongly indicates that interpersonal difficulties are an important aspect in the daily life of individuals with SZ.

In summary, our findings underscore the importance of analyzing FPAs using multiple methods to obtain converging evidence. We combined linguistic and network analyses to observe that individuals with or without interpersonal difficulties diverge in their language use that reflect their clinical symptoms.^59^ Personal narratives can reveal the complexity of living with SZ and the vast richness of their internal lives. Better understanding of these lives behind FPAs and integrating phenomenology into standard clinical care could lead to a more collaborative approaches to targeted treatments and, therefore, enhance therapeutic outcomes and a meaningful recovery.^60,61^

## Supplementary Material

sgag017_Supplementary_materials
