# Supplementary material for: Detecting Features of Interpersonal Difficulties in First-Person Accounts of Schizophrenia; Automated Linguistic and Network Analyses
Source: Schizophr Bull Open. 2026 Apr 30;7(1):sgag017. doi: 10.1093/schizbullopen/sgag017 (PMC13221957; doi:10.1093/schizbullopen/sgag017)
Supplement: sgag017_Supplementary_materials [file sgag017_supplementary_materials.zip › Supplementary_materials_sgag017_Table 1.docx]

**Supplementary Table 1.** Categorized Schizophrenia Bulletin First Person Accounts (1979-2025)

| **Year** | **Authors** | **Title** | **Article Category**  1 = First Person Account  2 = Third Person Account  3 = Poems/Artistic Expressions  4 = Researcher/Medical Professional Article  5 = Other Diagnosis | **Symptom Category**  0 = No Interpersonal difficulties  1 = Interpersonal difficulties | **Writer** | **Diagnosis** |
| --- | --- | --- | --- | --- | --- | --- |
| 1979 | DuVal, Martha | First person account: Giving love ….. and schizophrenia | 1 | 1 | First-Person | SSD |
| 1980 | Anonymous | First person account: After the funny farm | 1 | 0 | First-Person | SSD |
| 1981 | Minor, Dorothy | First person account: Third side of the coin | 1 | 0 | First-Person | SSD |
| 1981 | Anonymous | First person account: The quiet discrimination | 1 | 0 | First-Person | SSD |
| 1981 | Zelt, David | First person account: The Messiah quest | 2 | 0 | Clinician | SSD |
| 1981 | Anonymous | First person account: Problems of living with schizophrenia | 1 | 1 | First-Person | SSD |
| 1982 | Willis, Mary Jean | The Impact of Schizophrenia on Families: One Mother's Point of View | 2 | 0 | Mother | SSD |
| 1982 | Pringle, John & Pyke-Lees, Peggy | Voluntary Action by Relatives and Friends of Schizophrenia Sufferers in Britain | 2 | 0 | Father, Mother | SSD |
| 1982 | Peterson, Ronald | What Are the Needs of Chronic Mental Patients? | 1 | 1 | First-Person | SSD |
| 1982 | Loveyjoy, Marcia | Expectations and the Recovery Process | 1 | 0 | First-Person | SSD |
| 1982 | Houghton, Joan F. | First person account: Maintaining mental health in a turbulent world | 1 | 0 | First-Person | SSD |
| 1983 | Anonymous | First person account: A glimmer of light in the dark | 2 | 0 | Mother | SSD |
| 1983 | Brundage, Barbara E. | First Person Account: What I Wanted to Know But Was Afraid to Ask | 1 | 0 | First-Person | SSD |
| 1983 | Anonymous | First person account: A father's thoughts | 2 | 0 | Father | SSD |
| 1983 | Anonymous | First person account: Schizophrenia - pharmacy student's view | 1 | 1 | First-Person | SSD |
| 1984 | O'Neal, Jeanine M. | First Person Account: Finding Myself and Loving It | 1 | 0 | First-Person | SSD |
| 1984 | McGrath, Mary E. | First person account: Where did I go? | 1 | 0 | Firs-Person | SSD |
| 1984 | Lanquetot, Roxanne | First person account: Confessions of the daughter of a schizophrenic | 2 | 0 | Daughter | SSD |
| 1985 | Bockes, Zan | First Person Account: "Freedom" Means Knowing You Have a Choice | 1 | 0 | First-Person | SSD |
| 1985 | Stakes, Mark | First person account: Becoming seaworthy | 1 | 0 | First-Person | SSD |
| 1985 | Mittleman, Gladys | First person account: The pain of parenthood of the mentally ill | 2 | 0 | Mother | SSD |
| 1985 | Piercey, Bertha Peters | First person account: Making the best of it | 2 | 0 | Mother | SSD |
| 1986 | Fuchs, Lucy | First Person Account: Three Generations of Schizophrenia | 2 | 0 | Mother, Sister, Niece | SSD |
| 1986 | Slater, Eleanor | First person account: A parent's view on enforcing medication | 2 | 0 | Mother | SSD |
| 1987 | Woodman, Tim | First Person Account: A Pessimist's Progress | 1 | 1 | First-Person | SSD |
| 1987 | Ganim, Antoinette Rosa | First Person Account: The Delusion Girl--Diary of a Schizophrenic | 1 | 0 | First-Person | SSD |
| 1988 | Lanquetot, Roxanne | First Person Account: On Being Daughter and Mother | 2 | 0 | Mother, Daughter | SSD |
| 1988 | Fortner, Ross B. & Steel, Christine | First Person Account: The History and Outcome of My Encounter With Schizophrenia | 1 | 1 | First-Person | SSD |
| 1988 | Brodoff, Ami S. | First Person Account: Schizophrenia Through a Sister's Eyes--The Burden of Invisible Baggage | 2 | 0 | Sister | SSD |
| 1989 | Ruocchio, Patricia J. | First Person Account: Fighting the Fight--The Schizophrenic's Nightmare | 1 | 0 | First-Person | SSD |
| 1989 | Anonymous | First person account: How I've managed chronic mental illness | 1 | 1 | First-Person | SSD |
| 1989 | Crosby, Denise | First Person Account: Growing Up With a Schizophrenic Mother | 2 | 0 | Daughter | SSD |
| 1989 | Anonymous | First person account: A delicate balance | 1 | 0 | First-Person | SSD |
| 1990 | Caldwell-Smith, Gaetana | First person account: A mother's view | 2 | 0 | Mother | SSD |
| 1990 | Anonymous | First Person Account: A Pit of Confusion | 1 | 1 | First-Person | SSD |
| 1990 | Anonymous | First Person Account: Birds of a Psychic Feather | 1 | 1 | First-Person | SSD |
| 1990 | Anonymous | First Person Account: Behind the Mask: A Functional Schizophrenic Copes | 1 | 1 | First-Person | SSD |
| 1991 | Hyland, Betty | First person account: A thousand cloudy days | 2 | 0 | Mother | SSD |
| 1991 | Blaska, Betty | First person account: What it is like to be treated like a CMI | 1 | 0 | First-Person | SSD |
| 1991 | Smith, Evelyn | First person account: Living with schizophrenia | 2 | 0 | Mother | SSD |
| 1991 | Ruocchio, Patricia J. | First person account: The schizophrenic inside | 1 | 1 | First-Person | SSD |
| 1992 | Payne, Roberta L. | First person account: My schizophrenia | 1 | 0 | First-Person | SSD |
| 1992 | Stainsby, Jill | First person account: Schizophrenia: Some issues | 1 | 0 | First-Person | SSD |
| 1992 | Jaffe, Peter | First person account: My brother | 2 | 0 | Brother | SSD |
| 1992 | Anonymous | First person account: Portrait of a schizophrenic | 1 | 0 | First-Person | SSD |
| 1993 | Winship, Win | First person account: How do I let go? | 2 | 0 | Mother | SSD |
| 1993 | Turner, Barbara A. | First Person Account: The Children of Madness | 1 | 0 | First-Person | SSD |
| 1993 | Bowden, William D. | First Person Account: The Onset of Paranoia | 1 | 0 | First-Person | SSD |
| 1994 | Anonymous | **First person account: Schizophrenia with childhood onset** | 1 | 1 | First-Person | SSD |
| 1994 | DeMann, Jeffrey A. | First person account: The evolution of a person with schizophrenia | 1 | 1 | First-Person | SSD |
| 1994 | Anonymous | First person account: Life with a mentally ill spouse | 2 | 0 | Wife | SSD |
| 1994 | Gallo, Kathleen M. | First person account: Self-stigmatization | 1 | 0 | First-Person | SSD |
| 1995 | Fleshner, Chris L. | First person account: Insight from a schizophrenia patient with depression | 1 | 0 | First-Person | SSD |
| 1995 | Herrig, Elizabeth | First person account: A personal experience | 1 | 1 | First-Person | SSD |
| 1995 | Jordan, Janice C. | First person account: Schizophrenia -Adrift in an anchorless reality | 1 | 0 | First-Person | SSD |
| 1995 | Kagigebi, Alyce | First person account: Living in a nightmare | 2 | 0 | Mother | SSD |
| 1996 | Bayley, Robert | First person account: Schizophrenia | 1 | 1 | First-Person | SSD |
| 1996 | Brown, Georgia P. | First person account: Paranoid schizophrenia - sibling's story | 2 | 0 | Sister | SSD |
| 1996 | Wagner, Pamela Spiro | First person account: A voice from another closet | 1 | 0 | First-Person | SSD |
| 1996 | Anonymous | First person account: Social, economic, and medical effects of schizophrenia | 1 | 0 | First-Person | SSD |
| 1997 | Dykstra, Tracey | First person account: How I cope | 1 | 1 | First-Person | SSD |
| 1997 | Anonymous | First person account: The end of two roads | 1 | 0 | First-Person | SSD |
| 1997 | Murphy, Marcia A. | First person account: Meaning of psychoses | 1 | 1 | First-Person | SSD |
| 1997 | Molta, Victoria E. | First person account: Living with mental illness | 1 | 0 | First-Person | SSD |
| 1998 | Malloy, Ruth | First person account: My voyage through turbulence | 2 | 0 | Mother | SSD |
| 1998 | Powell, Jackie | First person account: Paranoid schizophrenia - A daughter's story | 2 | 0 | Daughter | SSD |
| 1998 | Bjorklund, Robert | First person account: Psychosocial implications of stigma caused by misdiagnosis | 5 | 0 | First-Person | Bipolar Disorder |
| 1998 | Lanquetot, Roxanne | First person account: A son recovered | 2 | 0 | Mother | SSD |
| 1999 | Hochman, Karen | First person account: Coming apart - tribute to Mark | 2 | 0 | Sister | SSD |
| 1999 | Mann, Susan B. | Talking Through Medication Issues: One Family's Experience | 1 | 1 | First-Person | SSD |
| 1999 | Sundquist, Amy | First person account: Family psychoeducation can change lives | 2 | 0 | Daughter | SSD |
| 1999 | “Hummingbird” | First Person Account: Schizophrenia, Substance Abuse, and HIV | 1 | 1 | First-Person | SSD |
| 2000 | Aldridge, S. L. | First person account: How the first wave of deinstitutionalization saved my mother from the "snake pit" | 2 | 0 | Daughter | SSD |
| 2000 | Greenblat, Leslie | First person account: Understanding health as a continuum | 1 | 0 | First-Person | SSD |
| 2000 | Chovil, Ian | First person account: I and I, dancing fool, challenge you the world to a duel | 1 | 1 | First-Person | SSD |
| 2000 | Campbell, Thomas | First person account: Falling on the pavement | 1 | 1 | First-Person | SSD |
| 2001 | Levin, Tomer | A Psychiatric Resident's Journey Through the Closed Ward | 4 | 0 | Psychiatric Resident | SSD |
| 2001 | Fox, Valerie | First person account: Schizophrenia, medication, and outpatient commitment | 1 | 0 | First-Person | SSD |
| 2001 | Ben-Dor, Sarah | Personal account | 2 | 0 | Mother | SSD |
| 2001 | Parker, Catherine | First person account: Landing a Mars lander | 1 | 1 | First-Person | SSD |
| 2002 | Fox, Valerie | First person account: A glimpse of schizophrenia | 1 | 1 | First-Person | SSD |
| 2002 | Anonymous (BGW) | Graduate student in peril: A first person account of schizophrenia | 1 | 1 | First-Person | SSD |
| 2002 | Chapman, Robert K. | First person account: Eliminating delusions | 2 | 0 | Friend | SSD |
| 2002 | Demers, Marie-France | First person account: Susan's cello | 1 | 0 | First-Person | SSD |
| 2003 | Weiner, Susan K. | First Person Account: Living With the Delusions and Effects of Schizophrenia | 1 | 1 | First-Person | SSD |
| 2003 | Salsman, Susan A. | First person account: The best medicine | 1 | 1 | First-Person | SSD |
| 2003 | Smith, Barbara B. | First person account: Medicines are not enough | 4 | 0 | Medical Professional | SSD |
| 2003 | Coleman, Roe Lynnette | Home sweet home | 1 | 1 | First-Person | SSD |
| 2004 | Schroeder, Cary | First Person Account: My Dream Life, a Normal Life | 1 | 0 | First-Person | SSD |
| 2004 | Fox, Valerie | First Person Account: Schizophrenia and Motherhood | 1 | 0 | First-Person | SSD |
| 2004 | Ribar, Allyson Varina | First Person Account: Schizoaffective Disorder and Suicide | 1 | 1 | First-Person | SSD |
| 2004 | Tolton, J. C. | How insight poetry helped me to overcome my illness | 1 | 0 | First-Person | SSD |
| 2004 | Sundstrom, Eric | First person account: The clogs | 1 | 0 | First-Person | SSD |
| 2005 | Lundin, Robert | The Mind Will Follow | 1 | 1 | First-Person | SSD |
| 2006 | Boevink, Wilma | From Being a Disorder to Dealing With Life: An Experiential Exploration of the Association Between Trauma and Psychosis | 1 | 1 | First-Person | SSD |
| 2006 | Stefanidis, Erin | Being Rational | 1 | 0 | First-Person | SSD |
| 2006 | Snyder, Kurt | Kurt Snyder’s Personal Experience with Schizophrenia | 1 | 0 | First-Person | SSD |
| 2007 | Fowler, Kristen | Snapshots: The First Symptoms of Psychosis | 1 | 0 | First-Person | SSD |
| 2007 | Anonymous | Why Having a Mental Illness Is Not Like Having Diabetes | 1 | 0 | First-Person | SSD |
| 2007 | Murphy, Marcia A. | Grand Rounds | 1 | 1 | First-Person | SSD |
| 2007 | Anonymous | Wayne S. Fenton, MD: A Patient’s Perspective | 4 | 0 | Medical Professional | SSD |
| 2007 | Chadwick, Peter K. | Peer-professional first-person account: Schizophrenia from the inside--Phenomenology and the integration of causes and meanings | 1 | 1 | First-Person | SSD |
| 2008 | Gray, Benjamin | Hidden Demons: A Personal Account of Hearing Voices and the Alternative of the Hearing Voices Movement | 1 | 1 | First-Person | SSD |
| 2009 | Rofe, Tzviel | Metaphorical Stories for Education About Mental Health Challenges and Stigma | 4 | 0 | Medical Professional | SSD |
| 2009 | Reina, Aaron | The Spectrum of Sanity and Insanity | 1 | 0 | First-Person | SSD |
| 2009 | Pushpa, K | Schizophrenia—A Victim’s Perspective | 1 | 0 | First-Person | SSD |
| 2009 | MacPherson, M | Psychological Causes of Schizophrenia | 1 | 0 | First-Person | SSD |
| 2009 | Kean, Clara | Silencing the Self: Schizophrenia as a Self-disturbance | 1 | 0 | First-Person | SSD |
| 2009 | Gray, Benjamin | Psychiatry and Oppression: A Personal Account of Compulsory Admission and Medical Treatment | 1 | 1 | First-Person | SSD |
| 2009 | Scotti, Paolo | Recovery as discovery | 1 | 1 | First-Person | SSD |
| 2010 | Anonymous | Taking the First Step | 1 | 0 | First-Person | SSD |
| 2010 | Puffer, A. Keith | The Intruder of the Mind | 2 | 0 | Son | SSD |
| 2010 | Greek, Milton | How a Series of Hallucinations Tells a Symbolic Story | 1 | 0 | First-Person | SSD |
| 2011 | Anonymous | Language Games | 1 | 0 | First-Person | SSD |
| 2011 | Adam* | Experiencing Suspicious Thoughts and Paranoia: An Account | 1 | 1 | First-Person | SSD |
| 2011 | Kean, Clara | Battling With the Life Instinct: The Paradox of the Self and Suicidal Behavior in Psychosis | 1 | 0 | First-Person | SSD |
| 2011 | West, Corinna | Powerful Choices: Peer Support and Individualized Medication Self-Determination | 1 | 0 | First-Person | SSD |
| 2011 | Rudnick, Abraham & Rofe, Tzviel & Virtzberg-Rofe, Daliah & Scotti, Paolo | Supported Reporting of First Person Accounts: Assisting People Who Have Mental Health Challenges in Writing and Publishing Reports About Their Lived Experience | 4 | 0 | Academic Researchers | SSD |
| 2012 | Payne, Roberta | Night’s End | 1 | 1 | First-Person | SSD |
| 2012 | Johnson, Amy | I Should Be Included in the Census | 1 | 1 | First-Person | SSD |
| 2012 | Jepson, Jason | The Sickness in Writing | 1 | 1 | First-Person | SSD |
| 2012 | Hawkes, Erin | Making Meaning | 1 | 0 | First-Person | SSD |
| 2012 | Fox, Joanna | Professional Discourse | 1 | 0 | First-Person | SSD |
| 2013 | Gray, Benjamin | Peer Support in Hospital—A Shared Journey | 1 | 0 | First-Person | SSD |
| 2013 | Timlett, Adam | Controlling Bizarre Delusions | 1 | 0 | First-Person | SSD |
| 2013 | Kauffman, Paul | A Carer’s Perspective: The View from Australia | 2 | 0 | Unspecified Relative | SSD |
| 2013 | Karlsson, Lis & Malmqvist, Annika | “Poetry in Yarn”—Making Sense of Life Experiences in the Shadow of Schizophrenia | 1 | 0 | First-Person | SSD |
| 2013 | Jepson, Jason | Teach Them to Be Self-Aware | 1 | 1 | First-Person | SSD |
| 2013 | Jepson, Jason | Bad Day | 1 | 0 | First-Person | SSD |
| 2013 | Jepson, Jason | When do you tell someone you are schizophrenic? | 1 | 0 | First-Person | SSD |
| 2014 | Humpston, Clara S. | Perplexity and Meaning: Toward a Phenomenological “Core” of Psychotic Experiences | 4 | 0 | Academic Researcher | SSD |
| 2014 | Payne, Roberta | Creativity and Schizophrenia | 1 | 0 | First-Person | SSD |
| 2014 | Reina, Aaron | Faith Within Atheism | 1 | 0 | First-Person | SSD |
| 2014 | Chadwick, Peter K. | Peer-professional first person account: Before psychosis-Schizoid personality from the inside | 1 | 1 | First-Person | SSD |
| 2014 | Bruni, Christina | Immediate intervention: Life-long success | 1 | 0 | First-Person | SSD |
| 2014 | Weiner, Susan | For Wayne S. Fenton, in Memoriam | 1 | 0 | First-Person | SSD |
| 2015 | Vitasola, Francesco | Why Am I Studying Neuroscience When I Have a Disease Science Can’t Explain: A Brief Synopsis From My Book Life Insane: My Memoir—Making of the Madman | 1 | 0 | First-Person | SSD |
| 2015 | Paul, Jay | The Vacuum of the Mind: A Self-Report on the Phenomenology of Autistic, Obsessive-Compulsive, and Depressive Comorbidity | 5 | 0 | First-Person | Autism Spectrum Disorder, Obsessive Compulsive Disorder |
| 2015 | Payne, Roberta | My Outsider Art | 1 | 0 | First-Person | SSD |
| 2015 | Watson, Molly | Listening to the Wherewho: A Lived Experience of Schizophrenia | 1 | 0 | First-Person | SSD |
| 2015 | Johnson, Amy | How understanding neuroscience helps me get unstuck | 1 | 0 | First-Person | SSD |
| 2016 | Paul, Jay | Understimulation of Cerebellum in Asperger’s Syndrome: A Personal Perspective | 5 | 0 | First Person | Autism Spectrum Disorder, Obsessive Compulsive Disorder |
| 2016 | Royal, Berenice | Schizophrenia: Nutrition and Alternative Treatment Approaches | 1 | 1 | First-Person | SSD |
| 2016 | Blanke, Christine & Bill, George | The Second World | 1 | 1 | First-Person | SSD |
| 2016 | Weiner, Susan | Six Pillars of Health | 1 | 0 | First-Person | SSD |
| 2016 | Palit, Sukanchan | Schizophrenia and Vision of My Life | 1 | 0 | First-Person | SSD |
| 2016 | Jepson, Jason | Acceptance | 1 | 1 | First-Person | SSD |
| 2016 | Jepson, Jason | A Vets Recovery | 1 | 0 | First-Person | SSD |
| 2016 | Gray, Benjamin | Recovery Champions: A Personal View on Making Recovery Happen | 1 | 0 | First-Person | SSD |
| 2016 | Ellerby, Mark | Schizophrenia, Maslow’s Hierarchy, and Compassion-Focused Therapy | 1 | 0 | First-Person | SSD |
| 2016 | Delbridge, Walter | Isolation and Intellect—A Letter and Selected Works by Walter K. Delbridge | 3 | 0 | First-Person | SSD |
| 2016 | Anonymous | Video and Poor Insight in Persons With Schizophrenia | 2 | 0 | Brother | SSD |
| 2016 | Hanley, Dominic | The journey | 1 | 1 | First-Person | SSD |
| 2016 | Helman, Daniel S. | Schizophrenia is normal: My journey through diagnosis, treatment, and recovery | 1 | 0 | First-Person | SSD |
| 2016 | Anonymous | The importance of talk therapy | 1 | 0 | First-Person | SSD |
| 2016 | Jepson, Jason | Relating to a schizophrenic | 1 | 0 | First-Person | SSD |
| 2017 | Yelser, Bethany | My Triumph Over Psychosis: A Journey From Schizophrenia and Homelessness to College Graduate | 1 | 0 | First-Person | SSD |
| 2017 | Meijer, May-May | "Mum, You Will Get Better”: Coping With a Vulnerability for Psychoses and Mania | 1 | 1 | First-Person | SSD |
| 2017 | Meijer, May-May | #PeaceAndLove: The Second Phase of My Psychoses | 1 | 1 | First-Person | SSD |
| 2017 | Beattle, Louise | Experiences of a First-Episode Psychosis by a Psychology Graduate Student | 1 | 0 | First-Person | SSD |
| 2017 | Colori, Steve | Understanding Referential Thinking | 1 | 1 | First-Person | SSD |
| 2017 | Colori, Steve | Autobibliotherapy | 1 | 1 | First-Person | SSD |
| 2017 | Jepson, Jason | Senses or Schizophrenia | 3 | 0 | First-Person | SSD |
| 2017 | Jepson, Jason | How Do You Know If You Are in La La Land? | 1 | 0 | First-Person | SSD |
| 2017 | Jepson, Jason | My Stages of Recovery | 1 | 1 | First-Person | SSD |
| 2017 | Fox, Joanna | The Contribution of Experiential Wisdom to the Development of the Mental Health | 1 | 0 | First-Person | SSD |
| 2017 | Ellerby, Mark | Resisting Voices Through Finding Our Own Compassionate Voice | 1 | 0 | First-Person | SSD |
| 2017 | Desousa, Avinash | Living with Schizophrenia | 3 | 0 | First-Person | SSD |
| 2017 | van der Pol, Annuska | Using Cognitive Behavioral Therapy on the Term Schizophrenia | 1 | 0 | First-Person | SSD |
| 2017 | Carroll, Donald | Severely Schizophrenic and Successful? Yes, It’s Possible! | 1 | 1 | First-Person | SSD |
| 2017 | Anonymous | My Experience With Psychiatric Services | 1 | 0 | First-Person | SSD |
| 2017 | Anonymous | A Father's Illness | 2 | 0 | Son | SSD |
| 2017 | Allan, Stephanie | Early Intervention in First Episode Psychosis: A Service | 1 | 0 | First-Person | SSD |
| 2018 | SA | The “Healing Healer”? A Psychologist’s Personal Narrative of Psychosis and Early Intervention | 4 | 0 | Psychologist | SSD |
| 2018 | Meijer, May-May | In the Garden of Eden: The Content of My Psychoses | 1 | 0 | First-Person | SSD |
| 2018 | Anonymous | Thought action fusion | 1 | 1 | First-Person | SSD |
| 2018 | Weiner, Susan | The Details of Schizophrenia | 1 | 0 | First-Person | SSD |
| 2018 | Colori, Steve | Journaling as Therapy | 1 | 1 | First-Person | SSD |
| 2018 | Colori, Steve | Working While Rehabbing | 1 | 1 | First-Person | SSD |
| 2018 | Colori, Steve | Exposure Therapy | 1 | 1 | First-Person | SSD |
| 2018 | Colori, Steve | Fear, Faith, Hope, and Courage | 1 | 1 | First-Person | SSD |
| 2018 | Anonymous | Learning to Live With Schizoaffective Disorder: A Transformative Journey Toward | 1 | 0 | First-Person | SSD |
| 2018 | Stewart, Janet | Picking up the Pieces | 1 | 0 | First-Person | SSD |
| 2018 | Jepson, Jason | Surviving the Voices | 1 | 1 | First-Person | SSD |
| 2018 | Jepson, Jason | A Positive Detour | 1 | 0 | First-Person | SSD |
| 2018 | Jepson, Jason | The Hammer: Something to Ignore | 1 | 0 | First-Person | SSD |
| 2018 | Jepson, Jason | My Relationship With My Caregivers | 1 | 1 | First-Person | SSD |
| 2018 | Ellerby, Mark | Schizophrenia: Stigma and the Impact of Literature | 1 | 0 | First-Person | SSD |
| 2018 | Ellerby, Mark | Reducing Voices by Direct Dialogue | 1 | 0 | First-Person | SSD |
| 2018 | Colori, Steve | Adversity; sometimes a gift | 1 | 1 | First-Person | SSD |
| 2019 | Ruoss, Betty | From Failing Meds to the Ones That Worked | 1 | 1 | First-Person | SSD |
| 2019 | Akram, Umair | Everything at Once, or Nothing at All | 1 | 1 | First-Person | SSD |
| 2019 | Sips, Rob | Psychosis as a Dialectic of Aha- and Anti-Aha-Experiences | 1 | 0 | First-Person | SSD |
| 2019 | Meijer, May-May | “I Do Not Know Anything about Your Hospitalization”: The Need for Triage at Psychiatric Hospitals | 1 | 0 | First-Person | SSD |
| 2019 | Anonymous | Intrusive Thoughts, Impulses, and Schizoaffective Disorder | 1 | 1 | First-Person | SSD |
| 2019 | Vijn, Thomas | The Ideal Psychiatry—A Utopia? | 1 | 0 | First-Person | SSD |
| 2019 | Ponte, Katherine | Stigma, Meet Hope | 5 | 0 | First-Person | Bipolar Disorder |
| 2019 | Jepson, Jason | About the Experiences | 1 | 0 | First-Person | SSD |
| 2019 | Jepson, Jason | Motivation for Writing | 3 | 0 | First-Person | SSD |
| 2019 | Jepson, Jason | Avenues of Support | 1 | 0 | First-Person | SSD |
| 2019 | Jepson, Jason | Reflections | 1 | 0 | First-Person | SSD |
| 2019 | Jepson, Jason | How I Made the Decision Not to Have Children | 1 | 1 | First-Person | SSD |
| 2019 | Newhill, Christina E. | Personal account: One out of a hundred | 2 | 0 | Friend | SSD |
| 2019 | Colori, Steve | Rhetoric and Recovery | 1 | 1 | First-Person | SSD |
| 2019 | Colori, Steve | Organization’s Effects With Schizoaffective Disorder | 1 | 0 | First-Person | SSD |
| 2019 | Colori, Steve | Facing Fears; If Medication Runs Out | 1 | 0 | First-Person | SSD |
| 2019 | Colori, Steve | Adversity and Justice | 1 | 1 | First-Person | SSD |
| 2019 | Colori, Steve | Disclosing My Diagnosis | 1 | 1 | First-Person | SSD |
| 2019 | Colori, Steve | Cognitive Impairment in an Episode of Schizophrenia Spectrum Disorder | 1 | 1 |  |  |
| 2020 | Jepson, Jason | Never Mind… I Have Car Insurance | 1 | 1 | First-Person | SSD |
| 2020 | Meijer, May-May & Meijer, Femke | “Maybe He Was in Love With You?”: How to Talk With People in Psychosis | 1 | 0 | First-Person | SSD |
| 2020 | Colori, Steve | My Experience With Hallucinations | 1 | 1 | First-Person | SSD |
| 2020 | Nicholas, Fiona Erica | Inside My Head | 1 | 0 | First-Person | SSD |
| 2020 | Jepson, Jason | Pursuing Trust | 1 | 0 | First-Person | SSD |
| 2020 | Anonymous | Resolving Repression | 1 | 1 | First-Person | SSD |
| 2020 | Francis, Robert | On delusion and self-recognition: A phenomenological perspective from the diagnosed (and functional) | 1 | 0 | First-Person | SSD |
| 2020 | Colori, Steve | The meaning of my diagnosis | 1 | 1 | First-Person | SSD |
| 2020 | King, Nicola | The Mad Professor | 1 | 0 | First-Person | SSD |
| 2020 | Arya, R | The Bearing of the Joint Family Unit on the Diagnosis and Delay of Schizophrenia Treatment | 2 | 0 | Unspecified Relative | SSD |
| 2020 | Abraham, Sajit | Delusional | 1 | 1 | First-Person | SSD |
| 2020 | Lenssen, Jaime & Verhagen, Simone | Monitoring My Journey From Doctor, to Patient, to Doctor With Lived Experience | 5 | 0 | First-Person | Major Depressive Disorder |
| 2020 | Goulet, Leslie Clark | My 49-Year Recovery From Mental Illness | 1 | 0 | First-Person | SSD |
| 2020 | Gowda, Charan | Unbox Different World of Schizophrenia: The Secret Key | 1 | 0 | First-Person | SSD |
| 2020 | Lawn, Sharon | On Loneliness | 2 | 5 | Wife | SSD |
| 2020 | Lee, Jean | Personal Experiences With Auditory Verbal Hallucination and Extrasensory Perception | 1 | 0 | First-Person | SSD |
| 2020 | Meijer, May-May | “I Did Not Tell it to Anyone”: Please Pay Attention to the Children of Patients Suffering From Psychosis | 1 | 0 | First-Person | SSD |
| 2020 | Weiner, Susan | Getting Well Again | 1 | 3 | First-Person | SSD |
| 2020 | Xia, Ji | Onset Schizophrenia | 1 | 4 | First-Person | SSD |
| 2021 | Li, Don | Navigating Schizophrenia in College | 1 | 0 | First-Person | SSD |
| 2021 | Jepson, Jason | Benefits of Staying on Medication | 1 | 0 | First-Person | SSD |
| 2021 | Francis, Robert | A Matter of Choice? | 1 | 0 | First-Person | SSD |
| 2021 | Christin, Wendy Singleton | Absence: Schizophrenia, Schizoaffective Disorder, and Marriage | 1 | 1 | First-Person | SSD |
| 2021 | Gardiner, Janice | First Person Account From a Person in Jail With Schizoaffective Disorder | 1 | 1 | First-Person | SSD |
| 2021 | Jepson, Jason | Coping With My Delusions | 1 | 0 | First-Person | SSD |
| 2021 | Tian, Rudy | Serenity Was the Reason for the Disappearance of My Positive Symptoms | 1 | 0 | First-Person | SSD |
| 2021 | Fox, Joanna Ruth | Lived Experiences of Psychosis: Understanding the Gap Between Perception and Reality | 1 | 0 | First-Person | SSD |
| 2021 | Arner, Matthew | A Psychotic Experience | 1 | 1 | First-Person | SSD |
| 2021 | Anonymous | Emotion–Action–Fusion, Intrusive Thoughts, and Psychosis | 1 | 0 | First-Person | SSD |
| 2021 | Warren, Mark | Boots | 4 | 0 | Doctor | SSD |
| 2022 | Tian, Rudy | Three Puzzles Explainable With My Experience | 1 | 0 | First-Person | SSD |
| 2022 | Colori, Steve | Dynamics of Sharing Lived Experience | 1 | 1 | First-Person | SSD |
| 2022 | Beyer, Anna Cornelia | On How to Create Nice Voices and How to Overcome Stigma | 1 | 1 | First-Person | SSD |
| 2022 | Mørck, Helene Cæcilie | In the Belly of the Whale | 1 | 0 | First-Person | SSD |
| 2022 | Gregersen, Leif | The Onset of My Schizophrenia | 1 | 0 | First-Person | SSD |
| 2022 | Mørck, Helene Cæcilie | God’s Light—A Tale About Revelations | 1 | 0 | First-Person | SSD |
| 2022 | Beyer, Anna Cornelia | Lack of Communication and Integration as Cause of Schizophrenia | 1 | 1 | First-Person | SSD |
| 2022 | Mørck, Helene Cæcilie | There Is a Crack in the Fabric of Time | 1 | 0 | First-Person | SSD |
| 2022 | Weiner, Susan | Schizophrenia and the Self | 1 | 0 | First-Person | SSD |
| 2022 | Jepson, Jason | What Is Triggering My Stress? | 1 | 0 | First-Person | SSD |
| 2022 | Beyer, Anna Cornelia | Love, God, and Coming Out of Stigma | 1 | 1 | First-Person | SSD |
| 2022 | Larrauri, Carlos Alberto & Staglin, Brandon | Leading Science With Lived Experience | 1 | 0 | First-Person | SSD |
| 2022 | Payne, Roberta | Poems | 3 | 0 | First-Person | SSD |
| 2022 | Weiner, Susan | The Humor in Schizophrenia | 1 | 0 | First-Person | SSD |
| 2022 | Arner, Matthew | The Quest for Reality | 1 | 0 | First-Person | SSD |
| 2022 | Beyer, Anna Cornelia | Schizophrenia as a Spiritual Experience and The Power of Prayer and Love | 1 | 1 | First-Person | SSD |
| 2022 | Jepson, Jason | A Soldier Comes Home and a War With Schizophrenia Begins | 1 | 0 | First-Person | SSD |
| 2023 | Arner, Matthew | A Psychotic Experience | 1 | 0 | First-Person | SSD |
| 2023 | Pierce, Bradford | Transcranial Magnetic Stimulation—A Patient’s First-Person Account on the Treatment of Schizophrenia’s Negative Symptoms (Avolition) | 1 | 0 | First-Person | SSD |
| 2023 | Anonymous | An Evil Cradling: First-Person Account of Psychosis During Pregnancy | 1 | 0 | First-Person | SSD |
| 2023 | Anonymous | The Bearing of the Joint Family Unit on the Diagnosis and Delay of Schizophrenia  Treatment | 2 | 0 | Unspecific Relative | SSD |
| 2023 | Jepson, Jason | Facing the Mountain | 1 | 1 | First-Person | SSD |
| 2023 | Jepson, Jason | Understanding My Personal Schizophrenia | 1 | 0 | First-Person | SSD |
| 2023 | Jepson, Jason | Blinders for Coping With Schizophrenia | 1 | 0 | First-Person | SSD |
| 2023 | Jeppsson, Sofia | My Strategies for Dealing With Radical Psychotic Doubt: A Schizo-Something Philosopher’s Tale | 1 | 0 | First-Person | SSD |
| 2023 | Mørck, Helene Cæcilie | Awakening from Schizophrenia | 1 | 1 | First-Person | SSD |
| 2023 | Xia, Ji | Mental Illness and Mental Health in Nursing Homes | 1 | 1 | First-Person | SSD |
| 2023 | Xia, Ji | Who’s Unreachable? | 1 | 0 | First-Person | SSD |
| 2024 | Gray, Ben & Sisto, Matthew | Hearing Voices Sessions in Providing Peer Support: A First-Person Account and Voice Hearer’s Perspective | 4 | 0 | Academic Researcher | SSD |
| 2024 | Lodge, Paul | Pyrrhonism” as a Therapeutic Response to the Allure of Mania | 5 | 0 | First-Person | Bipolar Disorder |
| 2024 | Regonini, Domenico | Coping With the Inner Turbulence | 1 | 0 | First-Person | SSD |
| 2024 | Dickson, Michael | From Vexing Uncertainty to Intellectual Humility | 1 | 0 | First-Person | SSD |
| 2024 | Guha, Sumit | Cognitive Impairment in an Episode of Schizophrenia Spectrum Disorder | 1 | 1 | First-Person | SSD |
| 2024 | Jespon, Jason | What do the Voices Say? | 1 | 2 | First-Person | SSD |
| 2024 | Jepson, Jason | Sharing My Paranoid Thinking | 1 | 0 | First-Person | SSD |
| 2024 | Ponte, Katherine | A Kind Gesture in the Psych Ward | 5 | 0 | First-Person | Bipolar Disorder |
| 2024 | Rafiuddin, Mohammed Sbahuddin & Sami, Musa Basseer | The Role of Religion and Ethnic Factors in My Recovery From 10 Years of Schizophrenia and Severe Depression | 1 | 0 | First-Person | SSD |
| 2024 | Weiner, Susan | Schizophrenia and the Self | 1 | 3 | First-Person | SSD |
| 2025 | Delgaram-Nejad, Oliver | Insight as a Barrier to the Diagnosis and Treatment of Mental Illness | 1 | 0 | First-Person | SSD |
| 2025 | Regonini, Domenico | Living With Schizoaffective Disorder: Healthier Ways to Cope With Anxiety, Fear, and Depression | 1 | 0 | First-Person | SSD |
| 2025 | Xia, Ji | Completely Delusional | 1 | 0 | First-Person | SSD |

***Note***. Article categories coded according to the following: 1 = First Person Account, 2 = Third Person Account, 3 = Poems/Artistic Expressions, 4 = Researcher/Medical Professional Article, 5 = Other Diagnosis; Interpersonal difficulties: negative syndrome identified using Schizotypal Personality Questionnaire (Raine, 1991). SSD = schizophrenia spectrum disorder.
