# Supplementary material for: Detecting Features of Interpersonal Difficulties in First-Person Accounts of Schizophrenia; Automated Linguistic and Network Analyses
Source: Schizophr Bull Open. 2026 Apr 30;7(1):sgag017. doi: 10.1093/schizbullopen/sgag017 (PMC13221957; doi:10.1093/schizbullopen/sgag017)
Supplement: sgag017_Supplementary_materials [file sgag017_supplementary_materials.zip › Supplementary_materials_sgag017_Table 2.docx]

**Supplementary Table 2.** Interpersonal Difficulties: Frequency and Examples

| **Article** | **Frequency** | **Example** |
| --- | --- | --- |
| Abraham (2020) Delusional | 4 | "The girls try to avoid me as much as possible" |
| Adam (2011) Experiencing Suspicious Thoughts and Paranoia: An account | 5 | "I felt quite lonely and isolated at school" |
| Akram (2019) Everything at Once, or Nothing at All. | 3 | “After the death of a close friend, additional thoughts relating to guilt and shame ensued along with the feeling of isolation" |
| Anonymous (1981) Problems of Living With Schizophrenia | 3 | "I felt so different and unattached to the world" |
| Anonymous (1990) A Pit of Confusion | 1 | "I have to learn to interact with people". |
| Anonymous (1990) Behind the Mask | 2 | "I did not talk about these things, so the only noticeable signs of my illness were that I became silent and withdrawn, not my usual ebullient and smiling self" |
| Anonymous (1994) Schizophrenia with Childhood Onset | 3 | "I want friends, but I don't know how to make them” |
| Anonymous (2018) Thought Action Fusion | 3 | "I experimented by thinking I can move into the person next to me and then would intentionally not move into them." |
| Anonymous (2019) Intrusive Thoughts, Impulses, and Schizoaffective Disorder | 1 | "I sought isolation to try liberating my mind from anything violent, sexual, or even the slightest bit unscrupulous" |
| Anonymous (2019) Resolving Repression | 2 | "I had psychosis and neurosis and I had lost all my friends and I was out of touch with my family" |
| Anonymous (1990) Birds of a Psychic Feather | 3 | "Some of my friends stopped speaking to me after the breakdown.” |
| Anonymous (1989) How I’ve Managed Chronic Mental Illness | 5 | "I was alone in my apartment, isolated from the world, and very depressed over the emptiness that was my life." |
| Anonymous (1983) Schizophrenia: A Pharmacy Student's view | 3 | "it's hard for me to talk to people" |
| Anonymous BGW (2002) Graduate Student in Peril | 2 | "I had a very small assortment of friends" |
| Arner (2021) A Psychotic Experience | 1 | “Since then I’ve been trying to hold things together while living on my own.” |
| Bayley (1996) Schizophrenia | 6 | "It is marked by profound misery and isolation and also by societies' neglect and misunderstanding" |
| Beyer (2022) Lack of Communication | 7 | "When I am all alone in my room all the time, I hear voices" |
| Beyer (2022) Love, God, and Coming Out of Stigma | 4 | "I had schizophrenia since 2002. I have lived through stigma: the isolation, ridicule, and avoidance of people that is really painful." |
| Beyer (2022) On How to Create Nice Voices and How to Overcome Stigma | 5 | "That is the reason the patient becomes paranoid and withdrawn and is also a reason for suicide" |
| Beyer (2022) Schizophrenia as a Spiritual Experience | 2 | "I lived there very spartanically and lonely and starved myself for love." |
| Blanke & George (2016) The Second World | 5 | "I didn’t make contact with the World of my friends nor with that of the passers-by." |
| Boevink (2006) | 1 | "But it also allowed me to withdraw more and more into my own crazy world and to isolate myself from the world around me." |
| Campbell (2000) Falling on the Pavement | 1 | "My isolated existence, having no friends to visit and living alone in my apartment, made these restrictions worse." |
| Carroll (2017) Severely Schizophrenic and Successful? Yes, It's Possible! | 1 | "By age 28, I had gone through a divorce with my wife, suffered various other losses from both drinking and drug use, and I eventually left Hawaii penniless for my hometown of San Jose, California." |
| Chadwick (2014) Before Psychosis | 5 | "I also was a person who had no commitments other than to the life of the mind, so I became a nomadic type, a loner, a recluse." |
| Chadwick (2007) Schizophrenia from the Inside | 2 | "I was alone and now trusted no one (if indeed my capacity to trust people [particularly after school] had ever been very high)" |
| Chovil (2000) I and I, Dancing Fool, Challenge You the World to a Duel | 5 | "I gradually lost all my human relationships, first my girlfriend, then my immediate family, then friends and coworkers." |
| Christin (2021) Absence: Schizophrenia, Schizoaffective Disorder, and Marriage | 15 | "A step into the abyss of aloneness." |
| Coleman (2003) Home Sweet Home | 3 | Social isolation and rejection by mainstream society is very painful for most people with schizophrenia." |
| Colori (2017) Autobibliography | 2 | “a good portion of my life I usually never expressed my true thoughts and did my best to stay with the group for fear of loneliness from my past experiences of being estranged." |
| Colori (2017) Understanding Referential Thinking | 1 | "I also hadn’t done anything wrong but thought I must have since I had lost all my friends from my first episode and felt there must have been something I had done for this to occur which wasn’t the case." |
| Colori (2018) Adversity; Sometimes a Gift | 6 | "I was left at age 24 without a functional mind, a job, very few friends, and I was living at home in my parents, basement." |
| Colori (2018) Exposure Therapy | 1 | "This occurred because I had difficulty trusting anyone other than myself unless I could see the logic they were presenting me with." |
| Colori (2018) Fear, Faith, Hope, and Courage | 11 | The cause of losing the friends was having schizoaffective disorder, and that is something I may always have." |
| Colori (2018) Journaling as Therapy | 1 | "There was no social anxiety present during a lecture, but obviously in a social situation there was and I had social trauma from a middle school experience where I was picked on daily for about a year and a half straight." |
| Colori (2018) Working While Rehabbing | 3 | "I spent a great deal of time sitting on the couch watching TV and despairing over having schizoaffective disorder and the state of life it had created for me which was a friendless and lonely place where I had nothing going for me, was lethargic, and lame from inactivity." |
| Colori (2019) ​​Rhetoric and Recovery | 3 | "I had been isolated from the modern world for 5 years, having talked to barely anyone, and I needed to learn how to reassimilate into modern society" |
| Colori (2019) Adversity and Justice | 1 | “lose most of my friends, it put me out of touch with my family” |
| Colori (2019) Disclosing My Diagnosis | 3 | "Isolation and loneliness were one of the most difficult and painful parts" |
| Colori (2020) My Experience with Hallucinations | 3 | "Places where I have been forced to socialize like work, parties, and large social gatherings have the most stressful places for me causing more hallucinations." |
| Colori (2020) The Meaning of My Diagnosis | 2 | "There seemed to be a social custom that people didn’t want anything to do with those who had mental illness.", |
| Colori (2022) Dynamics of Sharing Lived Experience | 1 | "Having this validation is powerful as it helps people to continue sharing and also know that they are not alone and there are others who have been through the same experiences they have." |
| Delbridge & Tucker (2017) Isolation and Intellect | 1 | "I’m fortunate to be able to explain this much, considering the amount of conflict brought into my existence and the discrimination and oppression that society has levered on me". |
| DeMann (1994) The Evolution of a Person With Schizophrenia | 3 | "I started to withdraw socially. " |
| DuVal (1979) Giving Love ... and Schizophrenia | 3 | "Only parents who have experienced an involuntary separation from a dependent child can understand the overwhelming joy of being united again. " |
| Dykstra (1997) How I Cope | 3 | "My mind seemed to spin so fast with racing thoughts that I could not carry on a conversation with anyone, and I completely withdrew from others." |
| Fortner & Steel (1988) | 5 | "It was, he said, an illness that tends to make you isolate yourself, withdraw, and have paranoid feelings. " |
| Fox Valerie (2002) A Glimpse of Schizophrenia | 2 | "I had crossed the line of faith into the evil world of schizophrenia: A barren world." |
| Gardiner (2021) | 3 | "I am locked in my little room alone for 22 hours a day." |
| Gray (2008) Hidden Demons | 1 | "You’re alone," an insidious voice told me. " |
| Gray (2009) Psychiatry and Oppression | 5 | "People with mental health problems who hear voices or hallucinate want to be valued, as we all do, not feared and ostracized." |
| Guha (2024) Cognitive Impairment in an Episode of Schizophrenia | 1 | "connecting with people on any subject became very difficult" |
| Hanley (2016) The Journey | 11 | "I felt sad, lonely, and worthless. " |
| Herrig (1995) A Personal Experience | 5 | "I didn't participate in any extracurricular activities or have any close friends.” |
| Hummingbird (1999) Schizophrenia, Substance Abuse, and HIV | 3 | "My family refused to visit me because it only upset me more. " |
| Jepson (2017) My Stages of Recovery | 5 | "For those who made eye contact with me, they could see me the “Loner” in their head just like I can see them in my head. " |
| Jepson (2018) My Relationship with My Caregivers | 1 | "My parents encourage me not to live a solitary life, but they also understand that sometimes I have to have alone time. " |
| Jepson (2018) Surviving the Voices | 2 | "Some people with schizophrenia isolate themselves or become reclusive. I have to work at not doing this." |
| Jepson (2013) Teach Them Self-aware | 2 | "I assure you, it is possible to feel lonely in the psyche ward even when you are around people who have some of the same symptoms you have. " |
| Jepson (2016) Acceptance | 3 | “But I was still alone.” |
| Jepson (2012) The Sickness in Writing | 2 | "A writer with a mental illness like me can feel alone and misunderstood." |
| Jepson (2019) How I Made the Decision Not to Have Children | 1 | "Sometimes when I'm having a bad day, I isolate myself to cope with a symptom" |
| Jepson (2020) Never Mind… I Have Car Insurance | 1 | "I had stopped going to class because the voices in my head coming from 2 people or a crowd were making me feel inadequate and alienated." |
| Jepson (2023) Facing the Mountain | 1 | "Sometimes l visualize that mountain outside my Army barracks where I once felt alone and helpless, trying to control the impulses of my brain." |
| Jepson (2024) What do the Voices Say? | 2 | "I often want to isolate myself, because of the voices inside my head" |
| Johnson (2012) I Should Be Included | 6 | "Not being able to communicate my basic feelings, not identifying with another human being, and feeling completely alone in my experience are killing me." |
| Lundin (2005) The Mind Will Follow | 4 | "My social skills were impacted: few people liked me, many distrusted me, and if I had any poise, it left me.” |
| Lawn (2020) On Loneliness | 5 | "I’m striving for the relationship, hanging on to the parts of our life that aren’t about mental illness, that don’t pull me too close to the edge of utter loneliness." |
| Mann (1999) Talking Through Medication Issues: One Family's Experience | 2 | "The only symptoms of my illness they had seen thus far were limited social withdrawal and the exhaustion that marked my face when the voices went on too long." |
| Meijer (2017) Mum You'll Get Better | 4 | "My husband divorced from me, I hardly saw my son, I quit my job and felt lonely." |
| Meijer (2017) Peace and Love | 2 | "I felt a lot of times very lonely." "I was very disoriented because I did not have a watch and because I stayed in an isolation room. " |
| Murphy (2007) Grand Rounds | 2 | "I failed numerous attempts at employment and continued to be socially isolated. |
| Murphy (1997) Meaning of Psychoses | 1 | "I had few friends, and except for being on the tennis team, I withdrew socially." |
| Mørck (2023) Awakening from Schizophrenia | 1 | "At the wards, I was left alone and felt isolated and miserable, which left me feeling non human and invisible" |
| Nicholas (2019) Inside My Head | 1 | "As a child, I was a loner with self-esteem problems and was bullied into misery." |
| Parker (2001) Landing a Mars Lander | 10 | "I feel that the minimum require- ments of relationships in my life for my psychological health are not met. Translation: I feel lonely and isolated. " |
| Payne (2012) Night’s End | 2 | "I have lost track of the real world and my schizophrenic brain exists in isolation. |
| Peterson (1982) What are the needs of chronic mental patients? | 6 | "When you're all by yourself, you can feel really lonely, even though you can see lots of people on the streets. But you're still isolated and by yourself. " |
| Royal (2016) Schizophrenia: Nutrition and Alternative Treatment Approaches | 4 | "I had no friends, no partner, no contact to my family. " |
| Ruocchio (1991) The Schizophrenic Inside | 12 | "The abysmal aloneness I feel is only made worse by the physical closeness of someone with whom I am trying so desperately to connect. " |
| Ruoss (2019) From Failing Meds to the Ones That Worked | 1 | "I had no social life" |
| Salsman (2003) The Best Medicine | 2 | "Realizing that I was not alone was a revelation." |
| Scotti (2009) Recovery as Discovery | 2 | " I became afraid to take the subway, believing that I was being followed, and withdrew from everyone thinking that they were plotting against me." |
| Weiner (2003) Living with Delusions | 1 | "In response to those data I changed my name at the DMV, broke contact with my family, friends, and school, colored my hair to hide my identity, and gave away all my belongings to begin a new life." |
| Weiner (2020) Getting Well Again | 3 | "devolved into illness and broke from my family, friends, and school before anyone could stop me or retard the development of insanity in my brain" |
| Weiner (2024) Schizophrenia and the Self | 3 | "I began to avoid people altogether hoping to break the cycle of brutishness I seemed to find in my heart." |
| Woodman (1987) A Pesssimist's Progress | 9 | "This increased my loneliness and desperation, and I entered into a vicious circle of be- havior when anything I did seemed to intensify my feelings. " |
| Xia (2023) Onset Schizophrenia | 4 | "I was stranded and they, whoever they were, were outrageously intrusive |
| Xia (2023) Mental Illness and Mental Health in Nursing Homes | 1 | "At NH I had trouble finding and maintaining friendship because the staff there wouldn’t let me or anyone else do what friendship entails, such as giving and taking" |
