# Supplementary material for: Detecting Features of Interpersonal Difficulties in First-Person Accounts of Schizophrenia; Automated Linguistic and Network Analyses
Source: Schizophr Bull Open. 2026 Apr 30;7(1):sgag017. doi: 10.1093/schizbullopen/sgag017 (PMC13221957; doi:10.1093/schizbullopen/sgag017)
Supplement: sgag017_Supplementary_materials [file sgag017_supplementary_materials.zip › Supplementary_materials_sgag017_Table 3.docx]

**Supplementary Table 3.**  Interpersonal items from the Schizotypal Personality Questionnaire (SPQ)

| **SPQ Subscale** | **SPQ Items** |
| --- | --- |
| ***Excessive Social Anxiety*** | I sometimes avoid going to places where there will be many people because I will get anxious. |
|  | I get very nervous when I have to make polite conversation. |
|  | Do you ever get nervous when someone is walking behind you? |
|  | I get anxious when meeting people for the first time. |
|  | Do you often feel nervous when you are in a group of unfamiliar people? |
|  | I feel very uncomfortable in social situations involving unfamiliar people. |
|  | I would feel very anxious if I had to give a speech in front of a large group of people. |
|  | I feel very uneasy talking to people I do not know well. |
| ***No Close Friends*** | I have little interest in getting to know other people. |
|  | I prefer to keep myself to myself. |
|  | I am mostly quiet when with other people. |
|  | I find It hard to be emotionally close to other people. |
|  | Do you feel that there is no one you are really close to outside of your immediate family, or people you can confide in or talk to about personal problems? |
|  | Writing letters to friends is more trouble than it is worth. |
|  | I tend to keep in the background on social occasions. |
|  | I attach little importance to having close friends. |
|  | Do you feel that you cannot get "close" to people? |
| ***Constricted Affect*** | People sometimes find me aloof and distant. |
|  | I am not good at expressing my true feelings by the way I talk and look. |
|  | I rarely laugh and smile. |
|  | My "nonverbal" communication (smiling and nodding during a conversation) is not very good. |
|  | I am poor at returning social courtesies and gestures. |
|  | I tend to avoid eye contact when conversing with others. |
|  | I do not have an expressive and lively way of speaking. |
|  | I tend to keep my feelings to myself. |

***Note***. SPQ: Schizotypal Personality Questionnaire (Raine, 1991)
